# Multimodal Analgesia Provides Superior Postoperative Pain Control Following Orthopedic Surgery in Small-Breed Dogs

**DOI:** 10.3390/ani16060878

**Published:** 2026-03-11

**Authors:** Seung-Hyun Kim, Seungjo Park, Chun-Sik Bae

**Affiliations:** 1 KSH Surgical Animal Medical Center, Gwangju 61617, Republic of Korea; 2 Department of Veterinary Medical Imaging, College of Veterinary Medicine and BK21 FOUR Program, Chonnam National University, Gwangju 61186, Republic of Korea; sjpark@jnu.ac.kr; 3 Department of Veterinary Surgery, College of Veterinary Medicine, Chonnam National University, Gwangju 61186, Republic of Korea

**Keywords:** multimodal analgesia, orthopedic surgery, postoperative pain, canine, CMPS-SF, hydromorphone, tramadol–lidocaine–ketamine, veterinary anesthesia, small-breed dogs

## Abstract

Effective pain control after orthopedic surgery is essential in veterinary practice, particularly in small-breed dogs with low physiological reserves. This study aimed to compare the analgesic efficacy and tolerability of five postoperative pain protocols across nine surgical procedures. A retrospective analysis was conducted in 205 small-breed dogs (≤7 kg) undergoing orthopedic surgeries. Dogs were assigned to one of five analgesic protocols: (A) carprofen, (B) tramadol–lidocaine–ketamine continuous-rate infusion, (C) butorphanol continuous-rate infusion, (D) hydromorphone continuous-rate infusion, and (E) multimodal analgesia combining local anesthetics, hydromorphone, and meloxicam. Pain was assessed at 6, 12, 24, 48, and 72 h using the Glasgow Composite Measure Pain Scale—Short Form. Analgesic efficacy was evaluated using pain trajectories, area-under-the-curve analysis, and pain resolution rates, and adverse effects were recorded. Dogs receiving multimodal analgesia achieved the most rapid and sustained pain relief, with all patients reaching pain resolution by 48 h. Hydromorphone alone showed comparable efficacy but was associated with more adverse effects, while tramadol–lidocaine–ketamine showed delayed pain relief and the highest rate of severe side effects. Multimodal analgesia provides superior pain control with acceptable safety in small-breed dogs undergoing orthopedic surgery, supporting its use based on surgical invasiveness and individual patient response.

## Simple Summary

Orthopedic surgeries in small-breed dogs often cause significant postoperative pain. Providing effective pain relief is essential not only for recovery but also for animal welfare. In this study, we compared five common pain management protocols used after orthopedic procedures, including a comprehensive multimodal approach combining local anesthetics, opioids, and anti-inflammatory drugs. We assessed pain levels over 72 h in 205 small dogs using a behavioral pain scale. The results showed that dogs receiving multimodal analgesia had the fastest and most complete pain relief, with fewer serious side effects compared to those receiving only opioids or single-drug treatments. Our findings support the routine use of multimodal analgesia in small-breed dogs undergoing surgery, especially in more invasive procedures. This evidence-based approach can improve recovery outcomes and reduce suffering, guiding veterinarians to make better postoperative pain management decisions.

## 1. Introduction

Effective postoperative pain management plays a central role in optimizing surgical outcomes and reducing the risk of complications in both human and veterinary orthopedic patients. In small-breed dogs, who are commonly predisposed to musculoskeletal disorders such as luxating patellae and cranial cruciate ligament rupture, orthopedic surgery is frequently indicated [1,2]. These procedures often involve significant tissue disruption, bone manipulation, and postoperative immobility—all of which contribute to moderate to severe postoperative pain [3,4]. When not effectively addressed, acute pain can evolve into chronic maladaptive pain, leading to hyperalgesia, allodynia, and behavioral changes, which are particularly problematic in smaller patients with limited physiological reserves [5,6]. Equally critical, however, is ensuring that analgesic protocols achieve these benefits while minimizing adverse effects such as gastrointestinal, neurological, or cardiovascular complications. Thus, both analgesic efficacy and safety must be considered as co-primary goals in clinical decision-making.

Over the past decade, pain management in veterinary medicine has evolved dramatically. The once-standard single-agent approach is now being replaced with multimodal analgesia (MMA) strategies, which combine agents targeting different pain pathways to provide synergistic relief while minimizing adverse effects [1,7,8,9,10]. MMA is especially valuable in orthopedic surgeries due to their high nociceptive intensity and the potential for prolonged postoperative pain, delayed return to normal ambulation, and extended functional recovery. In human medicine, MMA protocols are integral to enhanced recovery after surgery (ERAS), and their application in veterinary surgery is expanding rapidly with encouraging results [8,9,10,11].

Veterinary studies and clinical guidelines have highlighted the efficacy of combining NSAIDs, opioids, local anesthetics, and NMDA antagonists to modulate both peripheral and central sensitization [1,12,13,14]. NSAIDs such as meloxicam and carprofen have been demonstrated to reduce inflammation and enhance postoperative recovery in dogs undergoing orthopedic procedures [4,15,16]. Additional studies comparing meloxicam, carprofen, and paracetamol showed similar analgesic efficacy for moderate pain control [3]. However, opioid monotherapy, such as with hydromorphone, though highly effective in reducing pain, carries adverse effects including bradycardia, hypothermia, and dysphoria, necessitating cautious titration [1,17].

Butorphanol, an opioid agonist–antagonist, provides sedative and mild analgesic effects but is characterized by a ceiling effect and relatively short duration of action. Tramadol, especially when combined with lidocaine and ketamine in CRI formulations such as TLK, has shown promise in suppressing central sensitization [18]. The ketamine component provides NMDA receptor–mediated antinociceptive effects by reducing central sensitization, while lidocaine exerts both antiarrhythmic properties and systemic as well as peripheral analgesic effects [7,9]. Nonetheless, their efficacy as monotherapies is limited, particularly in highly invasive procedures like bilateral FHNO or TPLO [7,8,9,19].

Despite these advancements, analgesic protocols vary widely across clinics and regions. Alarmingly, many veterinary practices continue to rely on NSAIDs or single-dose opioids postoperatively, underutilizing multimodal strategies [2]. This variability underscores the need for evidence-based, procedure-specific guidelines. Comparable underreporting exists in laboratory animal models, where analgesic documentation is often lacking [20].

Pain intensity in orthopedic surgery varies substantially by procedure. Quantitative assessments using the Glasgow Composite Measure Pain Scale—Short Form (CMPS-SF) have shown that highly invasive procedures such as bilateral tibial plateau leveling osteotomy (TPLO) and bilateral femoral head and neck ostectomy (FHNO) can produce mean postoperative pain scores exceeding 14–16/24 within the first 6 h, often remaining above the intervention threshold for 24–48 h without adequate analgesia. In contrast, less invasive surgeries such as unilateral patellar luxation correction or simple fracture repair may yield peak CMPS-SF scores in the 8–10/24 range, resolving more quickly under standard NSAID–opioid protocols. These differences highlight the necessity for tailoring analgesic intensity to the expected nociceptive burden, as a “one-size-fits-all” regimen risks under-treating high-pain cases or overmedicating low-pain cases [12].

However, few studies have systematically compared multimodal analgesia protocols across these surgeries using validated tools such as the CMPS-SF, creating a gap in clinical guidance [6,21]. The CMPS-SF is currently the most widely adopted and validated behavioral pain scoring tool in small-animal practice. It evaluates multiple domains—including vocalization, posture, mobility, and response to palpation—allowing for both objective and subjective assessment of pain. Its high inter-rater reliability and sensitivity to analgesic effects make it ideal for clinical use [5,21]. Its measurement properties have also been further supported by recent cross-cultural validation studies conducted in clinical postoperative settings [22].

This study retrospectively analyzes a clinical cohort of 205 small-breed dogs (≤7 kg) that underwent one of nine orthopedic surgeries: Simple Fracture Surgery, Pelvic Fracture Surgery, Unilateral Patellar Luxation Surgery, Bilateral Patellar Luxation Surgery, Unilateral TPLO, Modified TPLO, Bilateral TPLO, Unilateral FHNO, Bilateral FHNO. Each case was categorized into one of five postoperative analgesia regimens: Carprofen, TLK CRI (tramadol–lidocaine–ketamine), Butorphanol CRI, Hydromorphone CRI, Intraoperative local Lidocaine & Bupivacaine + Hydromorphone CRI + Meloxicam SC.

CMPS-SF scores were recorded at 6, 12, 24, 48, and 72 h postoperatively. By mapping the pain response longitudinally and comparing it across standardized groups, this study aims to (1) quantify the analgesic efficacy of each regimen, (2) characterize the type and incidence of adverse events, and (3) identify surgery-specific analgesic strategies that maximize pain relief while minimizing risk. By giving equal weight to efficacy and safety, this study aims to provide evidence-based recommendations for optimal postoperative pain management in small-breed canine orthopedic patients, supporting clinicians in selecting multimodal analgesia strategies tailored to both surgical invasiveness and individual patient variability, thereby improving welfare and accelerating recovery. To the authors’ knowledge, no previous study has simultaneously evaluated procedure-specific postoperative pain trajectories and safety profiles of multiple analgesic protocols exclusively in small-breed dogs using longitudinal CMPS-SF assessment.

## 2. Materials and Methods

### 2.1. Recruitment of Subjects

This retrospective observational cohort study analyzed a total of 205 small-breed dogs (≤7 kg) that underwent one of nine orthopedic surgical procedures at a single veterinary surgical referral center between October 2024 and October 2025. All surgical procedures and postoperative analgesic treatments were performed as part of routine clinical care, and no treatment allocation was made for research purposes. The present study represents a retrospective analysis of anonymized clinical records collected during routine clinical management. To ensure accurate assessment of postoperative pain responses and minimize confounding factors, dogs with conditions known to alter pain perception were excluded. These included additional orthopedic disorders, neurologic diseases (e.g., intervertebral disc disease), and chronic systemic illnesses such as cardiac disease, renal insufficiency, or endocrine disorders. If any such conditions were identified during the preoperative evaluation, surgical intervention was deferred until the underlying issue was adequately treated. Preoperative assessment was performed using a combination of hematological tests, including CBC (ProCyte Dx, IDEXX Laboratories, Westbrook, ME, USA), serum chemistry and CRP (IDEXX chemistry analyzer, IDEXX Laboratories, Westbrook, ME, USA), and CPL and BNP (V200, BioNote, Hwaseong-si, Republic of Korea), as well as thoracic and abdominal radiography, abdominal ultrasound, and echocardiography. All patients were monitored for at least 72 h postoperatively. Monitoring was performed either through continued hospitalization or scheduled outpatient recheck visits depending on the analgesic protocol and owner preference. Only cases with complete medical records, consistent CMPS-SF scoring over the postoperative period, and confirmed analgesic protocol documentation were included. Additionally, patient variables such as age (in months), sex, neuter status, breed, body weight, body condition score (BCS), and American Society of Anesthesiologists (ASA) physical status classification were also recorded. Perioperative anesthetic management followed standardized institutional protocols and did not differ systematically among analgesic groups. Intraoperative analgesia included the use of opioid support (primarily hydromorphone) administered intravenously in titrated doses according to anesthetic monitoring parameters and surgical stimulation. These intraoperative analgesic measures were applied consistently across patients and were not associated with postoperative analgesic group assignment. No regional nerve blocks or epidural anesthesia were performed in any patient during the study period.

### 2.2. Surgical Categories

Patients were categorized based on the type of orthopedic surgery performed, as well as whether the procedure was unilateral or bilateral. The nine procedures were as follows: Simple Fracture Surgery, Pelvic Fracture Surgery, Unilateral Patellar Luxation Surgery, Bilateral Patellar Luxation Surgery, Unilateral TPLO, Modified TPLO, Bilateral TPLO, Unilateral FHNO, Bilateral FHNO. In all cases, no additional surgical interventions beyond the primary procedures described were performed.

### 2.3. Analgesic Group Assignment

Patients were grouped according to the postoperative analgesia protocol administered, selected by the attending anesthesiologist based on clinical indication. The five analgesic protocols were: Group A: postoperative hospitalization and injectable analgesic protocols were recommended by the attending surgeon in all cases. However, due to owner-related constraints, primarily financial considerations, hospitalization was declined. These dogs were discharged after the initial postoperative monitoring period and managed with oral carprofen (Zoetis, Parsippany, NJ, USA) alone, with subsequent pain assessments performed during scheduled recheck visits., Group B: TLK CRI (Tramadol, Lidocaine, Ketamine continuous-rate infusion), Group C: Butorphanol CRI, Group D: Hydromorphone CRI, Group E: Intraoperative local Lidocaine & Bupivacaine + Hydromorphone CRI + Meloxicam SC. Analgesic dosages were adjusted within the predefined dosing ranges of each protocol based on individual analgesic response, level of sedation, and occurrence of adverse effects. In Group B, a tramadol–lidocaine–ketamine (TLK) CRI was administered with the following dose ranges: tramadol (Jeil Jeyak, Yongin-si, Republic of Korea), 0.1–0.3 mg/kg/h; lidocaine (Dai Han Pharm, Seoul, Republic of Korea), 0.6–2.0 mg/kg/h; and ketamine (Huons, Seongnam-si, Republic of Korea), 0.12–0.6 mg/kg/h. In Group C, butorphanol (Myung Moon, Seoul, Republic of Korea) was administered at 0.1–0.2 mg/kg/h via CRI. In Group D, hydromorphone (Hana Pharm, Seoul, Republic of Korea) was delivered as a CRI at 0.1–0.4 mg/kg/h. In Group E, lidocaine (Dai Han Pharm, Republic of Korea) (4 mg/kg) and bupivacaine (Hana Pharm, Republic of Korea) (1 mg/kg) were applied intraoperatively as a surgical site splash block prior to wound closure. These local anesthetics were administered for peripheral nociceptive control and were not intended to produce regional motor blockade. Postoperatively, hydromorphone (Hana Pharm, Republic of Korea) was administered as a continuous-rate infusion (0.1–0.3 mg/kg/h), and meloxicam (Boehringer Ingelheim, Ingelheim am Rhein, Germany) was given subcutaneously at 0.2 mg/kg every 24 h as part of a multimodal analgesic regimen. To enhance reproducibility, the actual starting CRI doses administered were summarized as mean ± SD. In Group B (TLK CRI), the starting doses were tramadol, 0.21 ± 0.05 mg/kg/h; lidocaine, 1.28 ± 0.34 mg/kg/h; and ketamine, 0.31 ± 0.09 mg/kg/h. In Group C, butorphanol CRI was initiated at 0.15 ± 0.03 mg/kg/h. In Group D, hydromorphone CRI was started at 0.24 ± 0.07 mg/kg/h. In Group E, the postoperative hydromorphone CRI dose was 0.2 ± 0.05 mg/kg/h. Dose adjustments were performed as clinically indicated; the above values reflect initial infusion settings. Across all groups, any interventions that might have caused pain or exerted additional analgesic effects were strictly avoided during the period of analgesic administration. If a CMPS-SF score reached or exceeded the intervention threshold (≥6), immediate reassessment was performed, and analgesic dose adjustments were implemented within the assigned protocol. Rescue interventions consisted of dose escalation or adjustment of the ongoing analgesic regimen rather than protocol crossover. Pain scores were re-evaluated after adjustment to ensure adequate analgesic response. This approach reflects routine clinical pain management practice, where analgesic dosing is escalated as needed while maintaining the predefined treatment protocol for comparative analysis. All such modifications were documented in the medical record and reflected in the longitudinal CMPS-SF dataset.

### 2.4. Pain Assessment

Pain was assessed using the Glasgow Composite Measure Pain Scale—Short Form (CMPS-SF), a validated behavioral-based pain assessment tool for patients. Each patient was scored at five postoperative timepoints: 6 h, 12 h, 24 h, 48 h, 72 h. Each assessment was conducted by trained veterinary technicians who were not involved in analgesic protocol selection and were unaware of the specific study hypotheses. Due to the clinical nature of the analgesic protocols, complete blinding was not feasible, particularly for Group A patients who did not receive continuous infusion therapy. Therefore, this study employed partial blinding, and evaluators were instructed to adhere strictly to standardized CMPS-SF scoring guidelines to minimize observer bias. Although a formal sedation scoring scale was not applied, evaluators were trained to differentiate between analgesia-related behavioral improvement and drug-induced sedation by assessing multiple CMPS-SF domains, including response to palpation, posture, vocalization, and overall demeanor. Sedation was recorded separately as an adverse effect and considered during interpretation of pain scores.

All patients were hospitalized and monitored for at least 12 h postoperatively. For dogs discharged after this initial monitoring period (primarily Group A), subsequent CMPS-SF assessments at 24, 48, and 72 h were performed during scheduled outpatient recheck visits conducted by trained veterinary staff. No owner-performed pain scoring was used for study data collection. No regional nerve blocks or epidural anesthesia were performed in this study. In Group E, lidocaine and bupivacaine were administered solely as a surgical site splash block and were not intended to produce motor blockade. Therefore, postoperative locomotion assessment was not influenced by residual regional anesthesia. CMPS-SF scoring was conducted according to validated guidelines without exclusion of any item. Evaluators were trained to interpret locomotion findings in conjunction with other behavioral domains (e.g., posture, response to palpation, and demeanor) to differentiate pain-related impairment from expected postoperative mechanical limitations.

### 2.5. Adverse Effects Monitoring and Classification

Postoperative adverse effects were systematically assessed for all patients over the 72 h postoperative observation period. Observations were performed by attending veterinary staff and recorded in patient charts according to standardized criteria. The adverse effect grading system was prospectively defined prior to data collection and consistently applied across all cases to enable valid intergroup comparisons.

Adverse effects were classified according to a modified veterinary adaptation of the Common Terminology Criteria for Adverse Events (CTCAE v5.0) as follows: None: No observable adverse effects were noted; no modification of the analgesic regimen required.

Mild: Minor adverse effects (e.g., mild sedation, reduced appetite) not requiring analgesic change. Moderate: Manageable effects (e.g., nausea, transient dysphoria, bradycardia) requiring ~25% dose reduction; resolved within 12–24 h. Severe: Severe or life-threatening effects (e.g., profound bradycardia, hypotension, marked dysphoria) requiring ≥50% dose reduction or immediate intervention. Adverse effect data were subsequently analyzed by analgesic group to assess the tolerability profile of each protocol.

### 2.6. Statistical Analysis

All statistical analyses were performed using Python (version 3.11.5) with validated scientific libraries. A comprehensive statistical framework was applied to evaluate temporal pain trajectories, cumulative analgesic efficacy, and safety outcomes. Descriptive statistics were reported as mean ± standard deviation (SD) for continuous variables and proportions for categorical variables. The Friedman test, a non-parametric repeated measures analysis, was used to assess longitudinal changes in CMPS-SF scores within each analgesic group over time. Where significant, post hoc pairwise comparisons were conducted using Bonferroni-adjusted Wilcoxon signed-rank tests to control for multiple testing.

To quantify overall postoperative pain burden, the area under the curve (AUC) for CMPS-SF scores was calculated for each patient using the trapezoidal rule. Normality of AUC distributions and homogeneity of variances were assessed using the Shapiro–Wilk and Levene’s tests, respectively, prior to parametric analyses. Normality testing of AUC values using the Shapiro–Wilk test yielded W = 0.960 (*p* < 0.001). Although statistical deviation from normality was detected, the large sample size (*n* = 205) and the known robustness of two-way ANOVA to mild non-normality justified the use of parametric analysis. A two-way ANOVA was performed to assess the main effects of Analgesic Group and Surgical Procedure on AUC values, as well as their interaction (Analgesic Group × Surgical Procedure). A predefined subgroup analysis was conducted for each surgical type to evaluate protocol-specific performance. Effect sizes (η^2^) were calculated for each main effect and interaction term and interpreted using the following thresholds: small ≥ 0.01, medium ≥ 0.06, large ≥ 0.14.

When the interaction term was significant, simple main effects analyses were conducted to examine differences between analgesic protocols within each surgical type and, conversely, differences between surgical types within each analgesic group. Post hoc pairwise comparisons for these simple effects were performed using Tukey’s Honest Significant Difference (HSD) test with Bonferroni correction. Interaction patterns were visualized with interaction plots, and significant pairwise results were tabulated for clarity.

Group-level differences in categorical outcomes, such as the proportion of dogs achieving pain resolution (defined as CMPS-SF < 6 at 48 h postoperatively), were evaluated using Chi-square tests. Where overall significance was detected, Bonferroni-adjusted residual analysis was applied to identify specific group contrasts. For continuous measures of resolution rate, one-way ANOVA with Tukey’s HSD post hoc tests was used to confirm the magnitude and direction of differences.

The distribution of postoperative adverse effect severity (None, Mild, Moderate, Severe) across treatment groups was analyzed using the Kruskal–Wallis H test, reflecting the ordinal nature of the data. Pairwise comparisons between groups were conducted using Dunn’s test with Bonferroni correction.

To integrate efficacy and safety into a single interpretive framework, pain resolution rates were compared directly with the incidence of moderate-to-severe or severe adverse effects using dual-axis visualizations. Incidences of severe adverse effects were assessed via Chi-square tests, and these safety outcomes were interpreted alongside corresponding efficacy measures to characterize the clinical benefit-to-risk balance for each protocol. All statistical tests were two-tailed, with significance thresholds indicated as follows: * *p* < 0.05, ** *p* < 0.01, *** *p* < 0.001, **** *p* < 0.0001. ‘ns’ denotes non-significant differences (*p* ≥ 0.05).

The complete dataset underlying all statistical analyses and reported results is provided in Supplementary Data S1 (Supplementary Materials).

## 3. Results

### 3.1. Patient Characteristics

A total of 205 small-breed dogs (≤7 kg) met the inclusion criteria and were retrospectively analyzed. Eight breeds were represented, with the majority being Pomeranian (*n* = 81, 39.5%), Maltese (*n* = 56, 27.3%), and Poodle (*n* = 35, 17.1%). The mean age at surgery was 55.1 months. Most patients were classified as ASA physical status I (*n* = 67, 32.7%) or II (*n* = 82, 40.0%), with the remainder ASA III (*n* = 56, 27.3%).

Surgical procedures performed included unilateral patellar luxation correction (*n* = 47, 22.9%), bilateral patellar luxation correction (*n* = 42, 20.5%), unilateral FHNO (*n* = 25, 12.2%), simple fracture repair (*n* = 24, 11.7%), unilateral TPLO (*n* = 24, 11.7%), bilateral FHNO (*n* = 15, 7.3%), modified TPLO (*n* = 14, 6.8%), bilateral TPLO (*n* = 8, 3.9%), and pelvic fracture reconstruction (*n* = 6, 2.9%).

**Figure 1:**
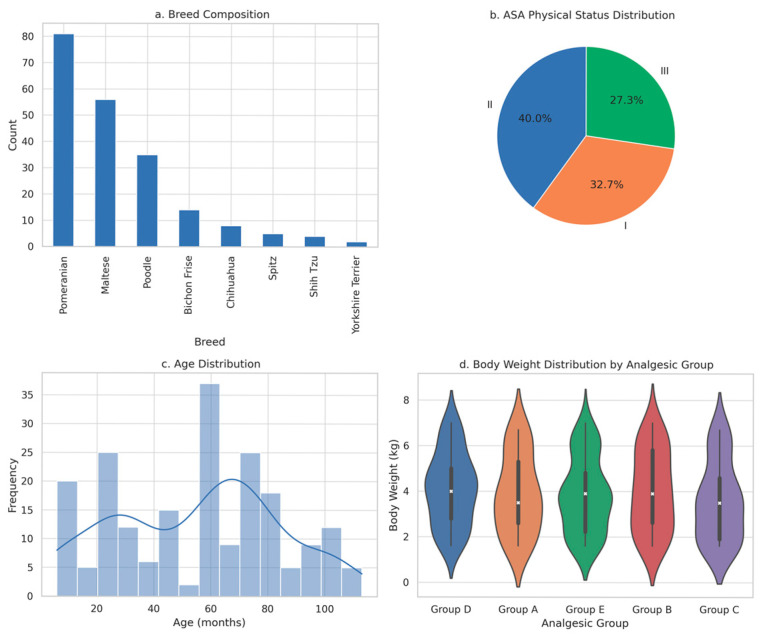
Baseline characteristics of the enrolled population are illustrated. (**a**) Breed Composition (**b**) ASA Physical Status Distribution (ASA, American Society of Anesthesiologists physical status classification; I = normal healthy patient, II = patient with mild systemic disease, III = patient with severe systemic disease) (**c**) Age Distribution (**d**) Body Weight Distribution by Analgesic Group (X indicates the mean body weight).

Postoperative analgesic protocol assignments were as follows: Group A (Carprofen; *n* = 25), Group B (TLK CRI; *n* = 34), Group C (Butorphanol CRI; *n* = 39), Group D (Hydromorphone CRI; *n* = 57), and Group E (Local lidocaine & bupivacaine + hydromorphone CRI + meloxicam SC; *n* = 50). Baseline characteristics, including age, sex, body weight, BCS, and ASA classification, showed no statistically significant differences among analgesic groups (all *p* ≥ 0.05), confirming comparability at study entry (Figure 1).

### 3.2. Temporal Patterns of Postoperative Pain

CMPS-SF scores were recorded at five postoperative timepoints (6, 12, 24, 48, and 72 h) for each surgical category, stratified by analgesic group (A–E). Across all surgical procedures, scores demonstrated a consistent downward trajectory over time, indicating progressive pain resolution. Friedman tests confirmed statistically significant changes over time in most groups and procedures (*p*  <  0.05 to *** *p*  <  0.0001).

**Figure 2:**
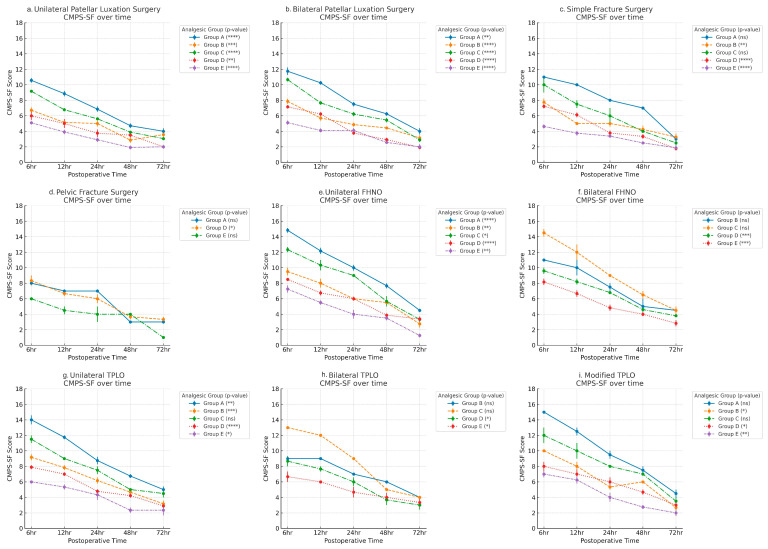
Temporal CMPS-SF score trends across analgesic groups for each surgical procedure. Each subplot represents a specific surgery type, showing mean ± SD CMPS-SF scores at 6 h, 12 h, 24 h, 48 h, and 72 h postoperatively. Statistical significance from Friedman test is denoted as follows: * *p* < 0.05, ** *p* < 0.01, *** *p* < 0.001, **** *p* < 0.0001, ns = not significant. (**a**) Unilateral Patellar luxation surgery (**b**) Bilateral Patellar luxation surgery (**c**) Simple Fracture surgery (**d**) Pelvic Fracture surgery (**e**) Unilateral FHNO (**f**) Bilateral FHNO (**g**) Unilateral TPLO (**h**) Bilateral TPLO (**i**) Modified TPLO.

Group E, employing a multimodal approach (local anesthetics, hydromorphone CRI, and meloxicam), consistently achieved the most rapid and substantial pain reduction, often approaching baseline pain scores by 48 h. In contrast, Group A (carprofen) maintained the highest scores at every timepoint, with many cases remaining in the moderate-to-severe pain range at 72 h, underscoring the necessity of more potent postoperative analgesia. Group B (TLK CRI) demonstrated moderate efficacy, outperforming Group A but generally lagging behind Groups D and E. Pain reduction was gradual, with more notable improvements after 24 h, especially in intermediate procedures such as unilateral TPLO. Group C (butorphanol CRI) exhibited variable results, performing comparably to Group B in low-to-moderate pain surgeries (e.g., patellar luxation) but showing inferior control in high-pain procedures such as TPLO and FHNO. Group D (hydromorphone CRI) consistently provided strong analgesia, particularly in high-pain surgeries like bilateral TPLO and FHNO, with rapid score reduction within 24 h. However, its effect was slightly less pronounced than the multimodal approach of Group E (Figure 2).

### 3.3. Postoperative Pain Burden Analysis by Analgesic Group

To comprehensively evaluate cumulative postoperative pain burden across analgesic protocols, we calculated the area under the curve (AUC) for each patient’s CMPS-SF scores measured at 6, 12, 24, 48, and 72 h postoperatively. AUC represents the integrated total pain experience, enabling robust comparisons between analgesic strategies while accounting for both intensity and duration of pain.

Group A (carprofen) exhibited the highest AUC values across all surgical categories, confirming the necessity of more potent analgesic intervention in postoperative care. Pain trajectories in this group showed delayed and incomplete resolution, with many patients remaining above the CMPS-SF clinical threshold even at 72 h. Group B (tramadol–lidocaine–ketamine CRI) demonstrated moderate pain control effectiveness, outperforming Group A but generally lagging behind opioid-based protocols. The onset of analgesia appeared slower, with more evident benefits emerging after 24 h. Group C (butorphanol CRI) presented intermediate results with high variability depending on the surgical procedure. It was effective in less invasive surgeries such as simple fractures or patellar luxation, but insufficient for high-pain procedures like bilateral TPLO or FHNO. Group D (hydromorphone CRI) achieved substantial reductions in total pain burden, maintaining consistently low CMPS-SF scores after the first 24 h. While effective, its performance was slightly inferior to the multimodal approach in Group E. Group E (multimodal analgesia) consistently demonstrated the most profound and rapid pain reduction, achieving near-baseline scores by 48 h in most surgical categories. This protocol’s integration of local anesthetics, opioids, and NSAIDs provided broad-spectrum analgesia addressing both peripheral and central pain mechanisms.

**Figure 3:**
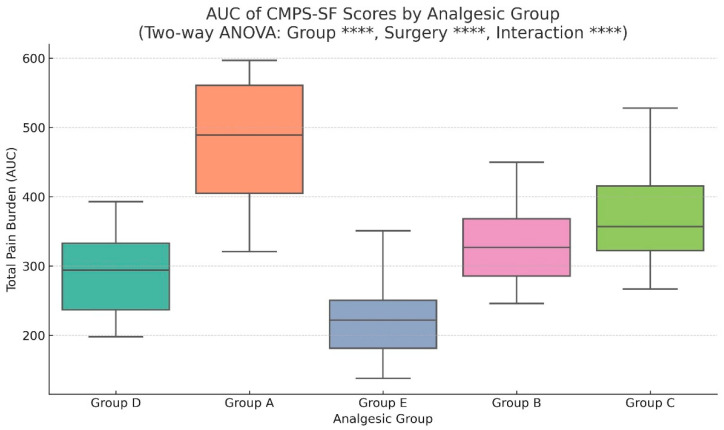
AUC of CMPS-SF Scores by Analgesic Group. Boxplots illustrate total pain burden (AUC) across the five analgesic protocols. Statistical significance (Two-way ANOVA) and η2 values are shown. Statistical significance is denoted as follows: **** *p* < 0.0001, ns = not significant. Group-wise differences highlight the superiority of multimodal analgesia (Group E) over other protocols.

Two-way ANOVA revealed highly significant main effects for both analgesic group (F = 503.44, *p* < 0.0001, ****, η^2^ = 0.329) and surgery type (F = 329.15, *p* < 0.0001, ****, η^2^ = 0.430), as well as a significant interaction effect (F = 41.06, *p* < 0.0001, ****, η^2^ = 0.215). According to Cohen’s benchmarks, these correspond to large effects for both main factors and a medium-to-large interaction effect, underscoring that analgesic efficacy is dependent on both the chosen protocol and the surgical invasiveness (Figure 3).

To address the potential confounding effect related to the non-hospitalized status of Group A, a secondary two-way ANOVA excluding Group A was performed. The main effects of analgesic group (F = 518.26, *p* < 0.0001) and surgery type (F = 105.28, *p* < 0.0001) remained highly significant. The interaction between analgesic group and surgery type also remained statistically significant (F = 3.26, *p* < 0.001). These findings confirm that the superiority of multimodal analgesia is not attributable solely to environmental differences associated with discharge status.

### 3.4. Effect Size Analysis of Analgesic and Surgical Factors

**Figure 4:**
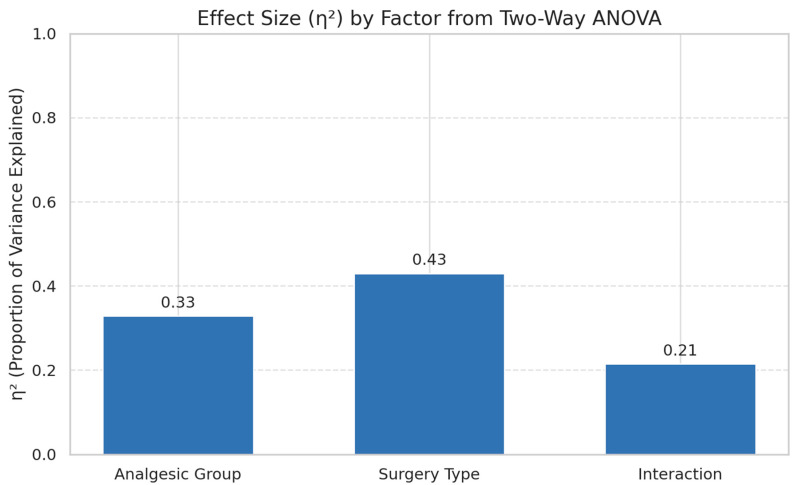
Effect size (η^2^) from two-way ANOVA showing the relative influence of analgesic protocol, surgery type, and their interaction on total pain burden (AUC). Large effect sizes were observed for all three factors, underscoring their clinical importance in multimodal pain management planning.

To quantify the relative contribution of analgesic protocol, surgical procedure, and their interaction to the variance observed in cumulative postoperative pain burden (AUC), effect sizes (η^2^) were calculated based on the two-way ANOVA model. Effect size magnitude was interpreted according to Cohen’s thresholds: small ≥ 0.01, medium ≥ 0.06, large ≥ 0.14. the surgical type accounted for the largest proportion of variance (η^2^ = 0.43, large effect), indicating that the nature of the procedure—likely reflecting differences in invasiveness, duration, and degree of tissue trauma—is the most dominant determinant of cumulative pain burden. The analgesic group explained a substantial portion of the variance (η^2^ = 0.33, large effect), confirming that pharmacological strategy significantly modulates postoperative pain trajectories regardless of surgical invasiveness. The interaction effect between surgical type and analgesic group contributed moderately to variance (η^2^ = 0.21, large effect), suggesting that the analgesic protocol’s effectiveness is not uniform across all surgical categories. This aligns with clinical intuition: certain multimodal regimens may be disproportionately beneficial for more invasive procedures, whereas simpler surgeries may achieve adequate pain control with less complex protocols. Taken together, these findings emphasize that while surgical invasiveness is the primary driver of postoperative pain burden, optimized analgesic selection tailored to the surgical context can further reduce pain substantially. The notable interaction effect supports a precision-medicine approach in veterinary analgesia—matching the protocol not just to the species and weight class, but to the anticipated pain intensity and recovery profile of the surgery itself (Figure 4).

### 3.5. Interaction Effects Between Surgery Type and Analgesic Group

To determine whether the analgesic protocol’s impact on postoperative pain varied by surgical procedure, a two-way ANOVA was performed with CMPS-SF scores as the dependent variable. The analysis identified highly significant main effects for both analgesic group (F = 542.84, *p* < 0.0001) and surgery type (F = 435.09, *p* < 0.0001), as well as a strong interaction effect between the two factors (F = 47.12, *p* < 0.0001). Effect size analysis revealed that the interaction term accounted for 21% of the total variance in pain scores (η^2^ = 0.21), which is considered a large effect. This magnitude underscores that analgesic efficacy is not uniform across surgeries; rather, the effectiveness of a given protocol depends strongly on the surgical context.

**Figure 5:**
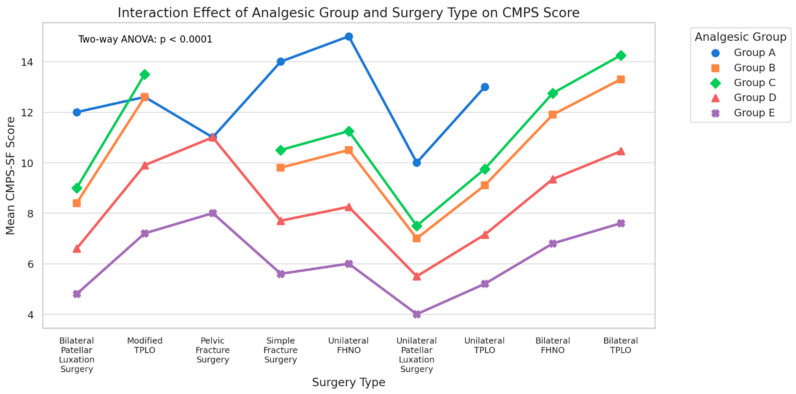
Interaction plot of mean CMPS-SF scores by analgesic group and surgery type. The significant interaction (*p* < 0.0001, η^2^ = 0.21) highlights substantial surgery-specific variation in analgesic efficacy.

Group E achieved the lowest CMPS-SF scores across most high-invasiveness surgeries (e.g., Modified TPLO, Bilateral FHNO), with absolute mean reductions exceeding 6–8 points compared to Group D. In low-to-moderate invasiveness surgeries (e.g., Unilateral Patellar Luxation), differences between Groups D and E were smaller, suggesting diminishing returns from multimodal regimens when nociceptive input is lower. Group A consistently produced the highest scores, confirming the detrimental impact of less-potent postoperative analgesia regardless of procedure type. Collectively, these findings provide robust evidence for tailoring analgesic protocols to the invasiveness, tissue trauma, and expected postoperative pain trajectory of each surgical procedure (Figure 5).

### 3.6. Pain Resolution at 48 Hours

**Figure 6:**
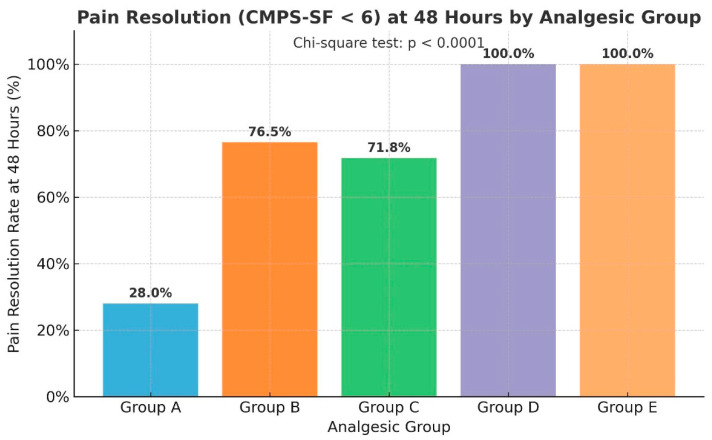
Pain resolution rates (CMPS-SF < 6) at 48 h by analgesic group.

Pain resolution was defined as achieving a CMPS-SF score < 6 at 48 h postoperatively. The proportion of dogs meeting this threshold varied markedly across analgesic groups. Specifically, Groups D and E achieved complete resolution (100%), while Groups B and C showed moderate resolution rates (76.5% and 71.8%, respectively). In contrast, only 28.0% of dogs in the Group A reached this level of pain relief. A Chi-square test confirmed that this difference in resolution rates was statistically significant (χ^2^ = 132.41, *p* < 0.0001), indicating a strong association between analgesic protocol and early postoperative pain control. Post hoc residual analysis revealed that Groups D and E significantly outperformed all other groups (adjusted *p* < 0.001), whereas Group A underperformed relative to expected rates (adjusted *p* < 0.001). These findings highlight that multimodal analgesia (Group E) and hydromorphone CRI monotherapy (Group D) provided rapid and complete pain resolution across diverse surgical types, while tramadol–lidocaine–ketamine (Group B) and butorphanol (Group C) CRI protocols delivered partial but clinically meaningful benefit (Figure 6).

### 3.7. Adverse Effects by Analgesic Group

Postoperative adverse effects were systematically evaluated across Groups B–E using a predefined ordinal grading scale (None, Mild, Moderate, Severe), applied consistently over the 72 h postoperative observation period. A total of 180 dogs were included in this tolerability assessment. Marked intergroup differences in adverse effect profiles were observed. Group B demonstrated the highest proportion of moderate-to-severe adverse effects, accounting for over two-thirds of cases, indicating a less favorable tolerability profile despite analgesic benefits. This elevated adverse event rate is likely attributable to the combined pharmacodynamic effects of tramadol, lidocaine, and ketamine, which can induce central nervous system excitation, dysphoria, and cardiovascular depression—effects that may be more pronounced in small-breed dogs with limited physiological reserves. Group C showed a predominance of none-to-mild adverse effects, reflecting a more favorable safety profile, albeit with moderate analgesic efficacy. The lower incidence of adverse events may be linked to butorphanol’s ceiling effect on respiratory depression and its relatively short half-life, limiting cumulative exposure. Groups D and E exhibited mixed profiles, with most patients experiencing mild or moderate effects, a low proportion of severe reactions, and some cases without any observable adverse events. In Group D, adverse effects such as bradycardia, hypothermia, and dysphoria were consistent with opioid-related pharmacology. In Group E, the incorporation of local anesthetics and NSAIDs likely reduced systemic opioid requirements, thereby lowering the incidence of severe events despite delivering high analgesic efficacy.

Across all groups, dogs with higher ASA status (II–III) tended to exhibit a greater incidence of moderate adverse effects, suggesting that baseline physiological resilience influences tolerability. The Kruskal–Wallis H test revealed a statistically significant difference in the distribution of adverse effect grades among the four groups (H = 88.73, *p* < 0.0001). Post hoc pairwise Dunn’s tests (Bonferroni-adjusted) indicated that Group B differed significantly from Groups C, D, and E, while no significant differences were observed between Groups C and E. From a clinical standpoint, these results underscore the importance of integrating tolerability outcomes into analgesic decision-making. While highly potent regimens may maximize pain relief, their adverse effect burden must be weighed carefully against the patient’s overall recovery trajectory.

To further detail the nature of these adverse events, the frequency of specific clinical signs observed within 72 h postoperatively was analyzed by analgesic group (Table 1). Group B demonstrated higher incidences of nausea (44.1%) and dysphoria (47.0%) compared to the other groups, along with a relatively increased occurrence of hypotension (23.5%). Group D showed the highest incidence of bradycardia (29.8%). Group C generally exhibited lower frequencies across most clinical categories, while Group E presented a balanced distribution without marked predominance of any single clinical sign. These findings clarify the clinical manifestations underlying the severity distribution observed among groups.

**Figure 7:**
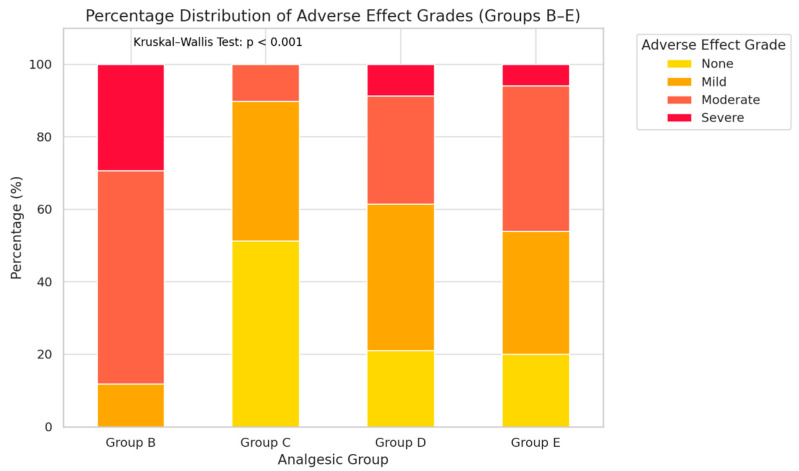
Percentage distribution of adverse effect grades across Groups B–E. Each stacked bar represents the proportion of dogs classified into four severity categories (None, Mild, Moderate, Severe). Significant intergroup variation was observed (Kruskal–Wallis H test, *p* < 0.001), with Group B demonstrating a notably higher incidence of moderate-to-severe adverse events compared to the other regimens.

Overall distribution of adverse effect severity is illustrated in Figure 7.

### 3.8. Analgesic Efficacy Versus Adverse Effects

**Figure 8:**
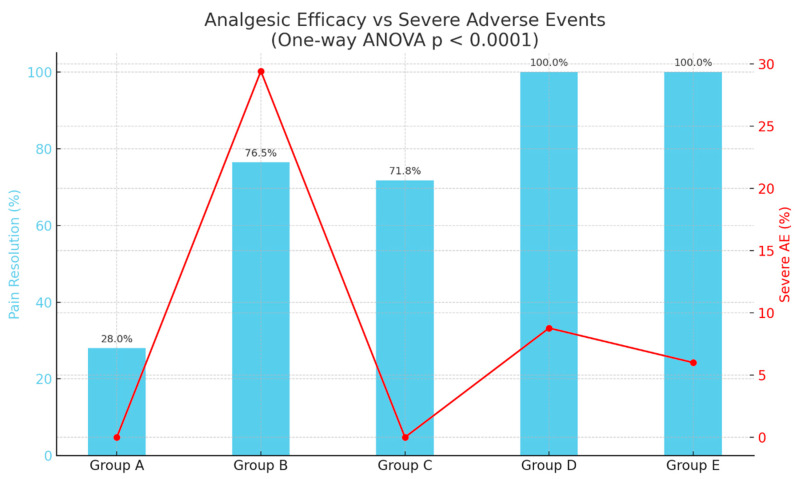
Comparison of analgesic efficacy (pain resolution rate at 48 h postoperatively, CMPS-SF < 6) and severe adverse event incidence across treatment groups. Blue bars depict mean resolution rates; the red line represents the proportion of dogs experiencing severe AEs. A one-way ANOVA indicated a significant difference in efficacy between groups (*p* < 0.0001).

To comprehensively assess the clinical trade-off between analgesic efficacy and treatment-related adverse effects, we jointly evaluated the proportion of dogs achieving pain resolution within 48 h postoperatively (CMPS-SF < 6) and the incidence of severe adverse events (AEs) across treatment groups (Figure 8). Pain resolution rates varied substantially: Group A (28.0%), Group B (76.5%), Group C (71.8%), and Groups D and E (100.0% each). One-way ANOVA on individual-level resolution data confirmed a statistically significant difference between groups (F = 29.56, *p* < 0.0001), underscoring marked heterogeneity in efficacy profiles. In terms of safety, severe AE incidence was highest in Group B (29.4%), followed by Group D (8.8%) and Group E (6.0%). Groups A and C exhibited no severe AEs (0.0%), indicating a superior tolerability profile.

From a clinical decision-making perspective: Group E provided maximal efficacy (100% resolution) while maintaining the lowest severe AE incidence, representing the most favorable benefit-to-risk ratio. The reduced opioid load due to local anesthetics and NSAIDs may have contributed to this balance. Group D matched Group E in efficacy but carried a slightly higher severe AE rate, likely reflecting dose-dependent opioid effects. Group C demonstrated intermediate efficacy with no severe AEs, making it a viable option in cases where safety concerns outweigh the need for maximal analgesia. Group B, despite achieving notable analgesia, had the highest severe AE incidence, possibly due to the cumulative sedative and cardio-depressant effects of the TLK combination, diminishing its clinical desirability.

These findings highlight the necessity of tailoring analgesic strategies not only for pain suppression but also for minimizing treatment-associated morbidity. Integrating both efficacy and safety data into the same analytical framework enables evidence-based, patient-specific postoperative analgesia planning (Figure 8).

## 4. Discussion

This retrospective cohort study systematically evaluated five postoperative analgesic protocols across nine distinct orthopedic procedures in small-breed dogs (≤7 kg). By employing the Glasgow Composite Measure Pain Scale—Short Form (CMPS-SF), a validated behavioral-based pain assessment tool [5,6], we quantified longitudinal pain trajectories and adverse event profiles for each regimen. Analysis of 205 cases revealed that multimodal analgesia (MMA)—specifically the protocol combining intraoperative local lidocaine and bupivacaine, continuous hydromorphone infusion, and subcutaneous meloxicam (Group E)—delivered the most rapid, profound, and sustained postoperative pain relief, with a markedly lower cumulative pain burden and a favorable safety profile compared to other protocols [12]. These results reinforce the principle that analgesic strategies should be both procedure-specific and patient-tailored, particularly in small-breed dogs with limited physiological reserves and heightened susceptibility to maladaptive pain responses [13,23]. A comprehensive statistical approach was intentionally employed to strengthen inference and offset the inherent limitations of a retrospective design.

Figure 1 illustrates that demographic and baseline surgical characteristics, including breed distribution, ASA classification, and weight, were balanced across all groups (*p* ≥ 0.05), minimizing baseline bias. The inclusion of nine surgical procedures, ranging from minimally invasive patellar luxation repair to highly invasive bilateral TPLO, enabled evaluation of analgesic efficacy across a broad spectrum of nociceptive intensities. Standardization of surgical technique by a single board-certified surgeon further strengthened the internal validity of comparative outcomes.

Pain trajectories assessed over 72 h (Figure 2) revealed consistent decreases in CMPS-SF scores for all active protocols, with Group E achieving the fastest and most complete resolution across all surgical types. Invasive surgeries such as bilateral TPLO and FHNO were associated with the highest baseline scores and required the most intensive analgesic strategies, while less invasive procedures such as fracture repair or unilateral patellar luxation responded adequately to intermediate protocols such as TLK CRI (Group B) or butorphanol CRI (Group C). The carprofen group (Group A) exhibited persistently high pain scores throughout the monitoring period, underscoring the clinical necessity for more potent analgesia postoperatively [2].

Integration of longitudinal scores into cumulative pain burden via AUC analysis (Figure 3) confirmed the superior performance of MMA (Group E), followed by hydromorphone CRI (Group D). Group A showed the highest total pain burden, and statistical modeling via two-way ANOVA identified large main effects for both analgesic group (η^2^ = 0.329) and surgery type (η^2^ = 0.430), as well as a large interaction effect (η^2^ = 0.215) (Figure 4). These effect sizes indicate that both analgesic protocol and surgical invasiveness independently and synergistically determine postoperative pain outcomes, supporting the need for individualized planning [12,24].

Importantly, effect size analysis revealed that surgical invasiveness (η^2^ = 0.43) exerted a greater influence on cumulative pain burden than analgesic protocol selection (η^2^ = 0.33). This finding suggests that while pharmacological strategy significantly modulates postoperative pain, the intrinsic nociceptive intensity of the procedure remains the primary determinant of overall pain trajectory. Therefore, analgesic planning should first stratify cases based on expected surgical invasiveness, followed by selection of an appropriately intensified multimodal regimen. The substantial interaction effect (η^2^ = 0.21) further supports a graded analgesic escalation model, whereby high-invasiveness procedures derive disproportionate benefit from multimodal strategies.

Interaction analysis (Figure 5) showed that analgesic efficacy varied significantly with surgical type. MMA provided substantial incremental benefit over opioid monotherapy for high-pain surgeries, whereas the advantage was less pronounced for low-pain procedures. This reinforces ERAS and veterinary guidelines emphasizing procedure-specific pain management [1,14]. From a clinical standpoint, these findings suggest that highly invasive procedures such as TPLO or FHNO may particularly benefit from a multimodal analgesic strategy, whereas less invasive procedures such as unilateral patellar luxation repair or simple fracture stabilization may achieve adequate postoperative pain control with less complex protocols when appropriate monitoring is maintained.

Pain resolution at 48 h (Figure 6) was universal in Groups D and E, while Groups B and C achieved resolution in approximately 70–77% of cases. The Group A reached only 28%, further demonstrating the critical role of more potent analgesia in achieving clinical recovery benchmarks.

Adverse event profiles (Figure 7) differed markedly between protocols. TLK CRI (Group B) was associated with a higher proportion of moderate-to-severe adverse effects. Butorphanol CRI (Group C) exhibited the lowest adverse event rate but provided limited analgesia, consistent with its known ceiling effect and short duration of action [25]. Hydromorphone CRI (Group D) was highly effective but carried typical opioid-associated risks, including dysphoria, bradycardia, and hypothermia [1,17]. MMA (Group E) combined high efficacy with a moderate adverse event incidence (~6% severe events), representing the most favorable benefit-to-risk profile. Importantly, despite combining multiple pharmacological agents, the multimodal protocol did not demonstrate an increased incidence of severe adverse events compared with the opioid-based protocols, suggesting that enhanced analgesic efficacy was achieved without compromising overall safety.

The integration of efficacy and safety (Figure 8) demonstrated that Group E offered the optimal clinical balance, achieving complete pain resolution with minimal severe adverse events. Group D, while equally effective in terms of pain control, showed a slightly higher rate of severe events, suggesting the need for careful patient selection. Group C emerged as a viable option for lower-pain surgeries or for patients where safety outweighs maximal analgesia, while the high adverse event rate in Group B limits its desirability despite reasonable analgesic performance.

The superiority of MMA observed here is mechanistically supported by the combined targeting of multiple nociceptive pathways. Lidocaine and bupivacaine provide preemptive peripheral blockade, meloxicam attenuates inflammatory mediator release, and hydromorphone modulates central perception of pain [24,26]. These complementary mechanisms likely underlie the reduced AUC and accelerated pain resolution observed in Group E.

This study has several limitations. Its retrospective design limits control over confounding factors such as perioperative stress, environmental conditions, and anesthetic depth. Although perioperative anesthetic management was standardized across patients and no regional nerve blocks or epidural anesthesia were performed, the retrospective design precludes complete exclusion of subtle confounding effects related to intraoperative analgesia or clinical decision-making. Restriction to small-breed dogs enhances applicability to toy and miniature breeds but limits extrapolation to larger breeds with different pharmacokinetics and pain behaviors. Although CMPS-SF is validated [21], it remains a semi-subjective tool and may underrepresent pain in stoic or anxious individuals. The 72 h observation window is adequate for acute pain assessment but does not capture subacute or chronic trajectories relevant to orthopedic recovery. An additional limitation of this study is that Group A differed structurally from the other analgesic groups, as these dogs did not undergo postoperative hospitalization or receive injectable analgesics due to owner-related constraints. Although this reflects real-world clinical decision-making, it may have introduced bias related to environmental factors and analgesic intensity, and therefore Group A should be interpreted as a minimal-analgesia reference group rather than a directly comparable cohort. An ethical consideration arises from the higher postoperative pain scores observed in Group A, particularly in more invasive surgical procedures. Although no analgesia was intentionally withheld and all treatments reflected real-world clinical decisions influenced by owner-related constraints, the increased proportion of dogs exceeding the intervention threshold underscores the limitations of NSAID monotherapy in certain orthopedic contexts. These findings highlight the importance of aligning analgesic intensity with expected surgical nociceptive burden to ensure optimal patient welfare in future clinical practice. Additionally, pharmacokinetic monitoring was not performed, limiting mechanistic interpretation, especially for tramadol-containing regimens where metabolic variability may influence efficacy [27]. Also, all surgeries were performed by a single experienced surgeon, which improves internal consistency but may limit external generalizability. Additionally, although partial blinding was implemented, the clinical differences between analgesic protocols may have introduced some degree of observer bias. Also, although sedation was documented as an adverse effect, the absence of a formal sedation scoring scale represents a methodological limitation of this study. Drug-induced sedation may suppress behavioral responses assessed by the CMPS-SF, potentially leading to underestimation of pain scores in some cases.

Future research should adopt prospective randomized controlled designs with broader breed inclusion, integrate objective recovery metrics such as accelerometry and gait analysis, and extend follow-up to capture long-term outcomes. Stratification by surgical invasiveness, comorbidities, and patient temperament would further refine personalized analgesic strategies.

In conclusion, the combined results confirm that multimodal analgesia using local anesthetics, opioids, and NSAIDs offers superior pain control and a balanced safety profile in small-breed dogs undergoing orthopedic surgery. Such evidence-based, procedure-specific protocols should be integrated into standardized postoperative care pathways to improve recovery quality and patient welfare.

## 5. Conclusions

This study clearly demonstrates that multimodal analgesia (MMA) outperforms single-agent protocols for postoperative pain management in small-breed dogs undergoing orthopedic surgery. The combination of local anesthetics, continuous hydromorphone infusion, and meloxicam (Group E) achieved the fastest and most sustained pain relief, the lowest cumulative pain burden, and 100% pain resolution within 48 h, while maintaining an acceptable safety profile. Tailoring analgesic strategies to surgical invasiveness and patient-specific factors is essential for optimal outcomes, and MMA should be considered a preferred postoperative analgesic strategy in this context. By integrating objective pain scoring with rigorous comparative analysis across diverse procedures, this work provides robust evidence to guide evidence-based analgesic protocols in small animal orthopedics. Future prospective, multicenter trials incorporating larger breed ranges, longer follow-up, and objective functional recovery metrics are warranted to confirm and extend these findings.

## Figures and Tables

**Table 1:** Frequency of Specific Clinical Signs within 72 Hours Postoperatively by Analgesic Group.

Clinical Sign	Group B (*n* = 34)	Group C (*n* = 39)	Group D (*n* = 57)	Group E (*n* = 50)
Sedation	13 (38.2%)	8 (20.5%)	15 (26.3%)	12 (24%)
Reduced appetite	8 (23.5%)	5 (12.8%)	11 (19.2%)	6 (12%)
Nausea	15 (44.1%)	2 (5.1%)	14 (24.5%)	4 (8%)
Dysphoria	16 (47%)	4 (10.2%)	10 (17.5%)	4 (8%)
Bradycardia	7 (20.5%)	3 (7.6%)	17 (29.8%)	8 (16%)
Hypothermia	2 (5.8%)	3 (7.6%)	8 (14%)	5 (10%)
Hypotension	8 (23.5%)	2 (5.1%)	4 (7%)	3 (6%)

Values are presented as number of dogs affected (percentage within group). Multiple signs could occur in the same patient.

## Data Availability

The complete dataset underlying all statistical analyses is available as Supplementary Data S1.

## Supplementary Materials

The following supporting information can be downloaded at: https://www.mdpi.com/article/10.3390/ani16060878/s1, Supplementary Data S1: Anonymized complete dataset underlying all statistical analyses and reported results.
